# Revealing the molecular relationship between type 2 diabetes and the metabolic changes induced by a very-low-carbohydrate low-fat ketogenic diet

**DOI:** 10.1186/1743-7075-7-88

**Published:** 2010-12-09

**Authors:** Judith Farrés, Albert Pujol, Mireia Coma, Jose Luis Ruiz, Jordi Naval, José Manuel Mas, Agustí Molins, Joan Fondevila, Patrick Aloy

**Affiliations:** 1 Anaxomics Biotech. C/Balmes 89, 08008 Barcelona, Spain; 2 Institute for Research in Biomedicine. Join IRB-BSC program in Computational Biology. C/Baldiri i Reixac 10-12, 08028 Barcelona, Spain; 3 Institute of Molecular Biology of Barcelona, Consejo Superior de Investigaciones Científicas, C/Baldiri i Reixac 10-12, 08028 Barcelona, Spain; 4 Asociación Médica Española de la Dieta Proteinada (AMEDPRO), C/Agustín Calvo 4, 28043 Madrid, Spain; 5 PronoKal, C/Roger Llúria 58, 08008, Barcelona, Spain; 6 Institució Catalana de Recerca i Estudis Avançats (ICREA), Pg. Lluís Companys 23, 08010 Barcelona, Spain

## Abstract

**Background:**

The prevalence of type 2 diabetes is increasing worldwide, accounting for 85-95% of all diagnosed cases of diabetes. Clinical trials provide evidence of benefits of low-carbohydrate ketogenic diets in terms of clinical outcomes on type 2 diabetes patients. However, the molecular events responsible for these improvements still remain unclear in spite of the high amount of knowledge on the primary mechanisms of both the diabetes and the metabolic state of ketosis. Molecular network analysis of conditions, diseases and treatments might provide new insights and help build a better understanding of clinical, metabolic and molecular relationships among physiological conditions. Accordingly, our aim is to reveal such a relationship between a ketogenic diet and type 2 diabetes through systems biology approaches.

**Methods:**

Our systemic approach is based on the creation and analyses of the cell networks representing the metabolic state in a very-low-carbohydrate low-fat ketogenic diet. This global view might help identify unnoticed relationships often overlooked in molecule or process-centered studies.

**Results:**

A strong relationship between the insulin resistance pathway and the ketosis main pathway was identified, providing a possible explanation for the improvement observed in clinical trials. Moreover, the map analyses permit the formulation of some hypothesis on functional relationships between the molecules involved in type 2 diabetes and induced ketosis, suggesting, for instance, a direct implication of glucose transporters or inflammatory processes. The molecular network analysis performed in the ketogenic-diet map, from the diabetes perspective, has provided insights on the potential mechanism of action, but also has opened new possibilities to study the applications of the ketogenic diet in other situations such as CNS or other metabolic dysfunctions.

## Background

### Novel approaches to study human disease networks

Efforts to link metabolic status or diseases by using simple, schematic biological pathways seldom offer the possibility to view the "broad picture", and are rarely able to explain the richness of the complex, redundant, intricate and sometimes blurry nature of human metabolism. Recently, the concept of "Diseaseome" [1] has arisen, as an essay to conceptualize at the highest level the relationship between observed phenotypes and underlying molecular and physiological disease mechanisms or disease cures. Although they are often treated separately, most human diseases are not independent of each other. Many diseases are associated with the breakdown of functional modules (subnetworks) of a complex network connecting many cellular components. Therefore, an understanding of the functionally relevant genetic, regulatory, metabolic and protein-protein interactions will play an important role in understanding the pathophysiology of human diseases [2].

Specifically, for studying human diseases from this perspective, one has to use a Systems Biology or Network Medicine approach [3-5], which are based on the construction of a complex network or map, where nodes are usually proteins, and edges are relationships between nodes. Relationships can be of any type: physical interaction between proteins, metabolic relationships and relationships driven by signaling pathways, homology or other characteristics. The physical representation of this concept is a cell network that can be queried and modeled to identify new complex pathways, or to identify previously unknown effectors of observable characteristics, like for example new drug targets or new metabolic relationships between mechanisms and pathways previously poorly understood [4,5].

In this work, we have applied these innovative concepts to suggest potential metabolic and molecular relationships between the observed clinical improvements in patients with type 2 diabetes and diet-induced ketosis.

### Ketogenic diets

Since ketosis is the physiological response to fasting, diets based on facilitating the ketosis status have been popular to promote weight loss. The commonly called ketogenic diets or very-low-carbohydrate diets (i.e. they restrict carbohydrate intake sufficiently to cause ketosis) include various diets that share in common a limit in carbohydrate intake while they vary in the relative distribution of protein, carbohydrate, and fat [6-8]. Low carbohydrate supply favors fatty acid oxidation, ketone body production, and their utilization as an alternative energy substrate, a metabolic state known as diet-induced ketosis [9]. These diets contribute to maintain the ketosis metabolic state during long periods of time, producing a rapid and substantial decrease in body weight in obese patients.

The benefits obtained from KD have been proposed as a treatment for several pathologies [10,11]. A KD with a high fat content has been used since 1920 for the treatment of difficult-to-control seizures in children [12]. Recently, several clinical trials have confirmed its efficacy [13]. Multiple theories have been proposed to explain how the KD protects against seizures [14]. Gene-expression profiling studies have also been used in order to identify the anticonvulsant mechanism of KD [15,16]. These studies have shown that the KD produces a coordinated up regulation of transcripts for genes encoding proteins involved in energy metabolism in rat hippocampus, including those specific to mitochondria.

Furthermore, ketone administration provides powerful neuroprotection in various CNS injury animal models [17] including: in vitro models of Alzheimer's [18] and Parkinson's disease [19], in vitro glutamate toxicity [20] and hypoxia [21]. All these intricate relationships highlight the huge connectivity observed between apparently unrelated biological processes which make systems approaches, to understand the global functioning of the individual, paramount.

### Ketogenic diets show beneficial effects on diabetic patients

Several clinical trials evaluating the effect of low-carbohydrate diets in patients with type 2 diabetes consistently showed improvements in glycemic control [22,23]. In fact, diets with a low charge of carbohydrates were used for the treatment of diabetes before insulin or other medication therapies were available [24,25].

In particular low-carbohydrate ketogenic diets have proved effective in improving glycemia and reducing medications in patients with type 2 diabetes. Yancy *et al*. in their study showed that Hemoglobin A1c (glycated hemoglobin (HbA1c), which is considered as an index of blood glucose control and the degree of oxidative stress in diabetes) decreased by 16% and diabetes medications were discontinued in 33% of the participants, reduced in 48% of the participants, and unchanged in 19% of the participants [26]. Because this effect occurs immediately upon implementing the dietary changes, individuals with type 2 diabetes who are unable to adjust their own medication or self-monitor their blood glucose should not make these dietary changes unless under close medical supervision. The data presented in the study of Dashti *et al*. showed that in addition to its therapeutic value, low carbohydrate diet is safe to use for a longer period of time in obese diabetics [27].

The improvements seen in the glycemic control of diabetic patients following this type of diets are not only attributable to a low-glycemic load as demonstrated in the study of Westman *et al *[28]. They compared a low-carbohydrate, ketogenic diet (LCKD) and a low-glycemic index diet (LGID) on diabetic patients. The LCKD led to greater improvements in glycemic control, and more frequent medication reduction/elimination than a low-glycemic index diet. In detail, the LCKD group had greater improvements in hemoglobin A1c (-1.5% vs. -0.5%, p = 0.03) and diabetes medications were reduced or eliminated in 95.2% of LCKD vs. 62% of LGID participants (p < 0.01). In fact, several authors point to a direct effect of the ketone bodies in the improvements seen in glucose levels [29,30].

Different variants of LCKD, like a very-low-carbohydrate low-fat ketogenic diet (protein-sparing modified fast, PSMF), have reported similar outcomes in diabetic patients [31].

Despite a history of clinical trials supporting the view that the LCKD has a significant beneficial effect on stabilizing hyperglycemia and ameliorating the diabetic state, the underlying mechanisms implicated in this protective effect are not understood. In this sense, our aim is to establish and evaluate the potential relationships, at molecular level, between the metabolic state of ketosis and type 2 diabetes, by means of a highly innovative network-based approach.

We have selected a very-low-carbohydrate low-fat ketogenic diet (PSMF) as the bases for the construction of the map as it promotes a non-atherogenic lipid profile compared to other type of ketogenic diets [32].

## Methods

### Construction of the ketosis status map

The first step in the construction of the cell network, or map, representing the metabolic state of ketosis due to protein-sparing modified fast consists in the identification of all those proteins described to be important in this particular metabolic state (i.e. seed proteins). With this purpose, and to make sure that we do not overlook any significant element, we have included in our analyses all molecules known to be related to the synthesis and degradation of ketone bodies as well as those involved in the amino acid metabolism, since it is known that PSMF diets have an increased proportion of protein respect to fat and carbohydrates. Pathway definitions were those of the Kyoto Encyclopedia of Genes and Genomes (KEGG) [33]. In particular, we selected all those molecules involved in the following physiological processes: hsa00071 Fatty acid metabolism; hsa00072 Synthesis and degradation of ketone bodies; hsa00100 Biosynthesis of steroids; hsa00230 Purine metabolism; hsa00380 Tryptophan metabolism; hsa00400 Phenylalanine; tyrosine and tryptophan biosynthesis; hsa00591 Linoleic acid metabolism; hsa00910 Nitrogen metabolism; hsa03320 PPAR signaling pathway; hsa04614; Renin-angiotensin system hsa04930 Type II diabetes mellitus). The map generation and extension process is conducted in recursive steps through the incorporation on the map of all known relationships of the seed proteins described using public data bases (KEGG, REACTOME, INTACT, BIOGRID) [33-36]. Relationships used to create the network can be of three main types: a) Physical interactions between proteins, known as interactome, and b) Metabolic relationships between proteins by sharing substrates, products and cofactors associated to chemical reactions, known as metabolome, and c) Relationships driven by signaling pathways, homology relationships or other characteristics.

### Analyzing the map

Anaxomics data base ax_SafetyDB is used to identify proteins related to different physiological conditions (syndrome, disease or pathological condition). It consists of hand-curated data base that contains more than 160 characterized conditions. The physiological pathways for each condition are identified and the proteins finally responsible of the phenotypical effect determined. The final responsible proteins for a phenotypical effect are called effector proteins. For instance, the insulin-like growth factor IB (IGF1) is a peptide hormone that is expressed in most tissues, it shares significant structural and functional similarities with insulin, and is implicated in the pathogenesis of insulin resistance and cardiovascular disease [37].

The final cell map is modeled as a direct connected graph, known as network, where nodes are usually proteins and edges (links) are relationships between nodes. The resulting graph is strongly connected and depicts complex cyclical structures. The pattern of connections of each node to the rest of the nodes of the graph can be seen as an "n" dimensional space with as many dimensions as number of nodes.

### Transforming the map into 2-dimensional images

For analyzing the relationship between proteins in the map, it is necessary to analyze the number and types of links between a given set of proteins. A conservative nonlinear projection method to visualize high-dimensional data into 2D has been applied. In this case a Multidimensional Scaling (MDS) transformation [38], is used to reduce the number of dimensions of the map into a 2 dimension (2D) to simplify the analysis. MDS transformation depicts closer those proteins that are closer (less number of links) in the original map, taking into account all possible ways to go from one to another surfing over the map. The color grading of images gives us an idea of the concentration of the different proteins in our maps (i.e. proteins/pixel^2^), from dark red (low protein concentration) to white (high protein concentration).

### Presence, centrality and clustering analysis

The relationship between two groups of proteins of interest (proteins related to ketosis and proteins related with type 2 diabetes) was assessed by means of standard measures in the field of biological networks, such as presence, centrality or clustering analysis [39].

Presence analysis evaluates the number of proteins present in the map in respect to the total number of known proteins associated to a physiological condition as described in Anaxomics private data base (ax_SafetyDB).

Centrality analysis evaluates if two groups of proteins are or are not homogeneously distributed over 2D transformation. The distance between groups was calculated by Hausdorff distance [40]. This type of measurements has been used before in systems biology field [41].

Clustering analysis allow the identification over 2D transformations of regions enriched in proteins with some remarkable characteristic, and is based on measures of protein density. The color grading of images gives an idea of the protein density, from dark blue (low protein density) to dark red (high protein density).

## Results

**Figure 1:**
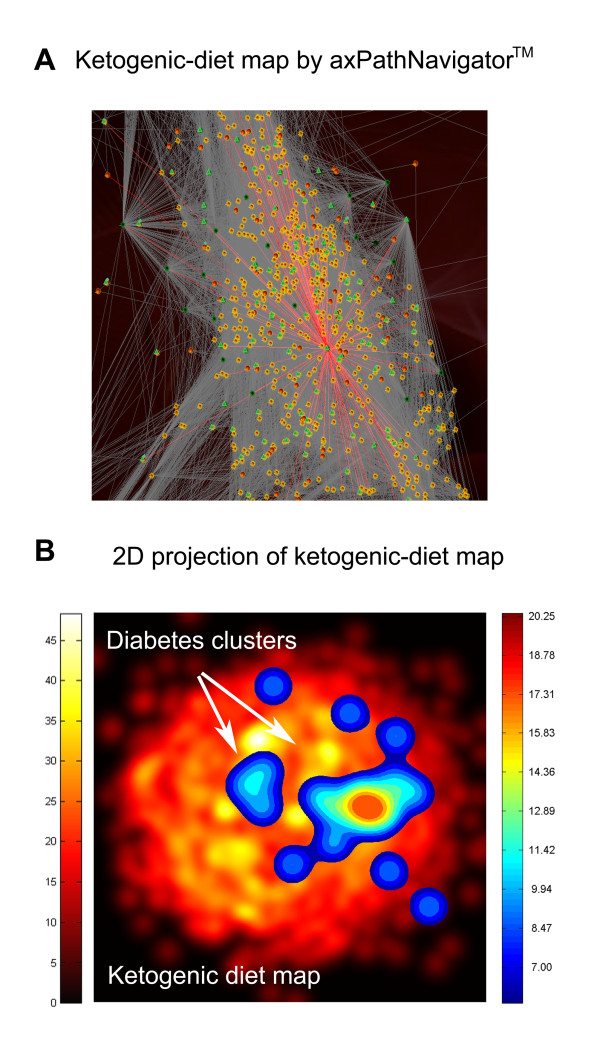
**Ketogenic diet map and type 2 diabetes**. **A) **Ketogenic-diet map by axPathNavigator™. Spheres represent proteins, lines are relationships between proteins and green triangles indicate proteins which are drug target. Seed proteins are colored in dark green. **B) **Diabetes clusters on 2D projection of ketogenic-diet map obtained through MDS transformation. The underling layer represents the ketogenic-diet map, the color grading of the image gives us an idea of the protein density, from black, no protein, until yellow areas of high protein density, see adjunct scale left (protein/pixel^2^). The overlaying image shows the location and density of the diabetes cluster on the ketogenic-diet map. Again, the color grading of the image gives us an idea of the protein density in this case from blue (low protein density) to dark red (high protein density), see adjunct scale right (protein/pixel^2^).

The ketosis map has been built by using 447 seed proteins from the ketosis pathway and proteins from other physiological pathways related to a protein-sparing modified fast (PSMF) a type of very-low-carbohydrate ketogenic diet as described in Methods. The final map includes 3,669 proteins and 142,531 known relationships. We will refer to it as the ketogenic-diet map (Figure 1A). Previous studies characterizing the effect of ketogenic diets (KD) on gene expression profiling in the hippocampus of rats identified up regulation of transcripts encoding oxidative phosphorylation and other mitochondrial proteins [15]. Some of the energy transcripts identified by Bought et al study were also reported in a previous microarray study of KD [16]. Most of the key gene identified in these gene expression profiling studies are also present in our map. From the 26 energy metabolism genes that were up regulated in the hippocampus of rats on a ketogenic diet, 18 have a human ortholog present in our map. Indicating that our map contains a comprehensive molecular description of the main effects related with the ketogenic state.

### Relationship at cell network level between the ketogenic-diet map and diabetes

The main objective of these analyses is to identify whether the molecular events responsible for diabetes are indeed present, in a significant manner, in the ketogenic-diet map. With this purpose, the relationship at cell network level between the metabolic state of ketosis and diabetes was assessed by presence, centrality and clustering analysis. Type 2 Diabetes, as characterized in ax_SafetyDB, includes 52 different effector proteins, 19 of which are present in the ketogenic-diet map, representing 36% of the total.

**Figure 2:**
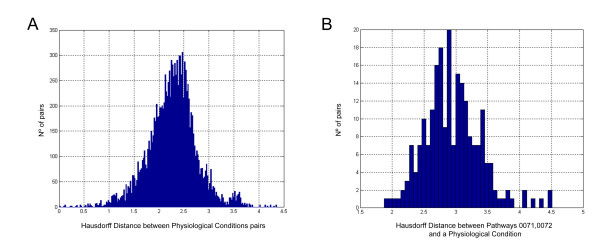
**Probability Density Function of Hausdorff distances**. The Hausdorff distance between any pair of physiological conditions is calculated as the average number of jumps between each protein contained in the first physiological condition against the closest proteins in the second physiological condition and vice versa. **A) **Probability Density Function of Hausdorff distances for any pair of physiological conditions described in ax_SafetyDB. This distribution shows the relation between all physiological conditions contained in ax_SafetyDB. It comprises a total of 12,882 comparisons that give a normal distribution with an average distance of 2.3 jumps and a standard deviation of 0.5 jumps. As can be seen in the distribution 95% of the measures are greater than 1.5 jumps. **B) **Probability Density Function of Hausdorff distances for all physiological conditions contained in ax_SafetyDB to the ketogenic pathways (path 71 and 72). It comprises a total of 228 comparisons. As can be seen in the distribution 95% of the measures are greater than 2.3 jumps.

Centrality analysis gives us an idea of the topological proximity at map level between diabetes and the ketosis status. The most representative proteins for each of the states are used for the measurement. The ketosis state induced by protein-sparing modified fast is represented by the 447 seed proteins, while diabetes is represented by the 19 effector proteins present in the map. The distance (Hausdorff distance) between the two groups of proteins of interest is of 1.5 jumps which is indicative of a high topological proximity between the two physiologic effects studied, type 2 diabetes and the ketosis status. To assess the statistical significance of this topological proximity, we calculated the general distribution of Hausdorff distances for each pair of physiological conditions contained in our database (Figure 2A), finding that a distance of 1.5 jumps, or fewer, is only observed by 5% of the motifs studied.

The clustering analysis identifies cores or groups of effector proteins of diabetes present in the ketogenic-diet map that are topologically relevant by MDS transformation (Figure 1B).

### Relationship at metabolic pathway level between the ketogenic-diet map and diabetes

The next step is to address whether the macromolecular components, constitutive of diabetes, identified in the ketogenic-diet map are indeed related to type 2 diabetes, as the clinical observations indicate. Accordingly, we conducted similar analyses to those described above but, this time, analyzing each of the type 2 diabetes pathophysiological pathways (motives) independently. Two different motives have been identified for type 2 diabetes in ax_SafetyDB: insufficient insulin production and insulin resistance.

The presence analysis indicates that the effector proteins related to insulin resistance motive are found in greatest amount in the ketogenic-diet map (13 representing a 56% of all effector proteins for this specific pathway). See Table 1 for details.

**Table 1:** Relationship at metabolic pathway level between the ketogenic-diet map and type 2 diabetes

Ketogenic-diet map	Pathophysiological pathways of Type 2 Diabetes	
***Presence***	***Insufficient insulin production***	***Insulin resistance***
% effector proteins present in ketogenic-diet map (No. protein present/Total No. in data base)	23% (7/30)	56% (13/23)
***Distance to ketogenic pathways (Hausdorff distance)***	***Insufficient insulin production***	***Insulin resistance***
Fatty acid metabolism (KEGG: HSA00071) (No. of jumps, minimum distance)	2.3 (0.95)	2.5 (0.85)
Synthesis and degradation of ketone bodies (KEGG: HSA00072) (No. of jumps, minimum distance)	2.3 (0.95)	2.5 (0.85)

Values in brackets report the significance of the distance value. It has been estimated from the distribution of the Hausdorff distances of all pathophysiological conditions contained in ax_SafetyDB to pathways (path 71 and 72). The same principles as the ones described in figure 2 have been applied.

The distance analysis between the proteins of the different KEGG pathways selected as seed proteins and the proteins of the two physiological pathways related to type 2 diabetes did not identify significant differences between the pathways analyzed (data not shown), since all KEGG pathways studied showed a close relationship to both type 2 diabetes motives. The beneficial effects of ketogenic diets observed in clinical studies on patients with type 2 diabetes has been typically attributed to the low carbohydrate content of this type of diets, and the concomitant weight loss. However, there is still a significant beneficial contribution that cannot be directly attributed to the previously mentioned aspects, and that some authors relate to the ketosis status [25,29,30]. In order to explore this hypothesis further, we have centered the analysis of distances and relationships at a protein level on the two main pathways of the ketosis state: the synthesis and degradation of ketone bodies and the fatty acid metabolism, and we left for coming projects exploration of the relationships with the other metabolic pathways used in the map construction.

The distance analysis of the two main pathways of the ketosis state used to construct the map (i.e. metabolism of ketone bodies and fatty acids) are significantly closer than what would be expected by chance to the pathophysiological pathways related to type 2 diabetes, with distances of 2.3 and 2.5 jumps, respectively (Table 2). The calculated probability distribution of the Hausdorff distances of all pathophysiological conditions contained in ax_SafetyDB to the main ketosis state patways (71 and 72) indicates that distances minor or equal to 2.5 jumps are only seen in 15% of the cases and a distance minor or equal to 2.3 jumps is only found in 5% of the cases (Figure 2B).

**Table 2:** Direct relationships (1 jump) between seed proteins of ketogenic-diet map and type 2 diabetes effector proteins.

Physiologic Pathway	Type 2 Diabetes Effector Protein (Protein Name)	*Gene Name (synonyms)*
Insufficient insulin production	Somatotropin	GH1

	Solute carrier family 2, facilitated glucose transporter member 4 (GLUT4)	SLC2A4 *(GLUT4)*
	
	E3 ubiquitin-protein ligase CBL (CBL)	CBL *(CBL2, RNF55)*
	
	Protein kinase C alpha type (PRKCA)	PRKCA *(PKCA, PRKACA)*
	
Insulin resistance	Nuclear factor NF-kappa-B p105 subunit (NFKβ)	NFKB1
	
	Insulin receptor substrate 1 (IRS-1)	IRS1
	Peroxisome proliferator-activated receptor gamma coactivator 1-alpha (PPARGC1-α)	PPARGC1A *(LEM6, PGC1, PGC1A, PPARGC)*
	
	Protein kinase Akt-3 (PKBγ)	AKT3 *(PKBG)*

**Figure 3:**
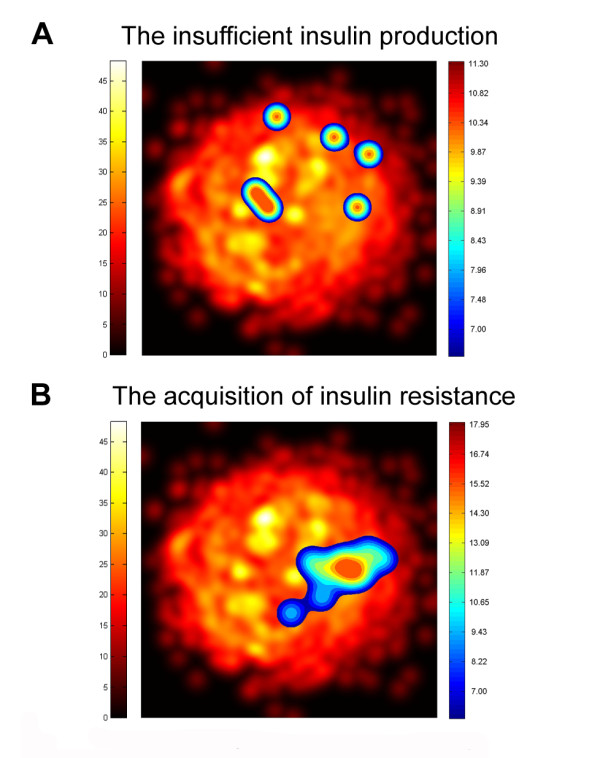
**Clusters of the different type 2 diabetes motifs on a 2D projection of ketogenic-diet map**. The underlying image represents the ketogenic-diet map obtained through MDS transformation. The color grading of the image gives us an idea of the protein density, from black, no protein, until yellow areas of high protein density. Overlaying this image the localization of the protein clusters for the different type 2 diabetes motifs is presented. The color grading of images gives us an idea of the protein density from dark red (high-density proteins) to blue (low-density of proteins) (protein/pixel^2^).

The existence of nuclei or sets of diabetes effector proteins from the two physiological pathways of type 2 diabetes that have a topological relationship on the map was also analyzed by clustering analysis. The highest level of clustering is found in proteins that define the route of insulin resistance (Figure 3), concluding thus that it is, indeed, this pathological process the one that is the most embedded within the ketogenic-diet map.

### Relationship at protein level between the ketogenic-diet map and diabetes

The analysis of the relationship between effector proteins of diabetes and seed proteins of ketogenic map was also conducted. We found a total of 371 relationships between seed proteins used to build the ketogenic-diet map and diabetes effector proteins. Of those relationships, 33 are direct, i.e., direct interaction established between 8 diabetes proteins and 30 seed proteins in the ketosis-map, notice that one protein can establish more than one relation. From the 33 direct relationships between type 2 diabetes and the seed proteins of the ketosis map, 30 (88%) correspond to relations with proteins of the insulin resistance pathway.

**Figure 4:**
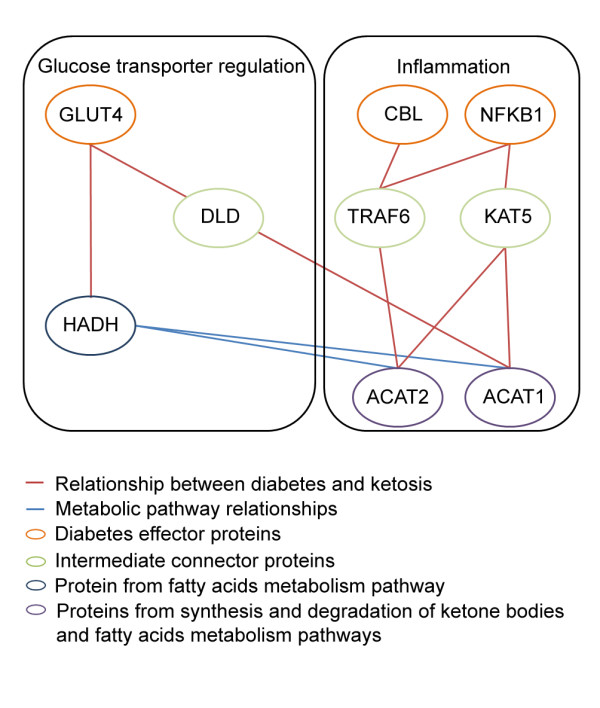
**Protein relationship between effector proteins involved in the insufficient insulin production motive of diabetes and the key pathways of the ketogenic diet**. Two complementary hypothesis of functional relationship between the molecules involved in both physiological processes have evolved: a) Elements of lipid metabolism may facilitate proper cellular localization of glucose transporter and recycling and b) Ketone bodies can alleviate certain inflammatory processes by blocking specific cytokines.

Among the 8 type 2 diabetes effector proteins which establish direct relationships, there are 7 from the insulin resistance pathway (SLC2A4, CBL, PRKCA, NFKB1, IRS1, PPARGC1A and AKT3) and 1 from the insufficient insulin production pathway (GH1), see details of the proteins involved in Table 2. From the 33 seed proteins establishing direct relationships with diabetes proteins we find 3 (ACAT1, ACOX1 and HADH1) from the central routes of ketosis, synthesis and degradation of ketone bodies and lipid metabolism. In summary, this analysis of direct interactions at the protein level indicates that type 2 diabetes motive, insulin resistance, is in close relationship with proteins in the ketosis pathway (Figure 4).

## Discussion

Clinical trials provide evidence of benefits of very-low-carbohydrate ketogenic diets in terms of clinical outcomes of type 2 diabetes patients. Recently, advances in systems biology and disease network analysis allow new approaches to understand and reveal the complex and intricate relationships between molecular network and different metabolic phenotypes. In this sense, our aim was to reveal such relationship between ketogenic diet and type 2 diabetes by means of systems biology methodologies, and specifically, by topological analysis of the diabetes effector proteins in the ketogenic diet molecular network map.

Our analysis suggests that, from the innovative perspective of the global topological analysis of molecular networks describing the two conditions, the relationship between the ketosis status induced by protein-sparing modified fast and type 2 diabetes is closer to the insulin resistance pathway than to insufficient insulin production. Furthermore, the analysis at protein level shows that 88% of the direct relationships between diabetes effectors and the ketogenic-diet map are done by proteins related to the insulin resistance pathway, while proteins related to insufficient insulin production are less present (Table 2).

Our studies at the protein level have identified that one of the effector proteins of the insulin resistance pathway, GLUT4, has a direct relation with proteins of the fatty acids metabolism (HADH1 and ACOX1) a key pathway of the ketogenic diet (Figure 3). GLUT4, Glucose transporter type 4, facilitates glucose transport into the cell in an insulin-responsive manner. Insulin increases intracellular glucose concentration by transferring GLUT4 to the plasma membrane localization where it exerts its function via vesicles that require acylated lipid. HADH, encodes for the enzyme 3-hydroxyacyl-coenzyme A dehydrogenase and catalyses the penultimate reaction in the beta-oxidation of fatty acids. Defects in HADH are the cause of familial hyperinsulinemic hypoglycemia type 4. It has been hypothesized that HADH deficiency causes modulation of other genes associated with the insulin secretion apparatus and thus triggers insulin secretion [42], however, the mechanism remains unclear [43].

Actually, the GLUT4- HADH interaction has been anticipated earlier and a role in priming fatty acids for protein acylation by adjusting acyl chain length by b-oxidation has been proposed [44]. The interaction may be part of the regulatory mechanisms that modulate the catalytic activity of GLUT4 or it could participate on the recycling between stocks and functional storage in the membrane.

On the other hand, the direct relationship of GLUT4 with ACOX1, Peroxisomal acylcoenzyme A oxidase 1, seems unfeasible as this enzyme is located in the peroxisome while GLUT4 resides in the cytoplasm or associated to the membrane.

The analysis at the molecular level also provided another provable way in which ketone bodies and insulin resistance can be related. We found CBL and NFKβ, two proteins related with insulin resistance and also with inflammation at two jumps distance from the mitochondrial (ACAT1) and cytosolic (ACAT2) form of acetyl-CoA acetiltransferase, an enzyme of lipid metabolism involved on the fatty acids metabolism and the synthesis and degradation of ketone bodies.

Conditions in which inflammatory cytokines are elevated insulin resistance has also been shown to occur [29]. Ketone bodies reportedly counteract certain inflammatory processes by blocking the proinflammatory cytokine, macrophage migration inhibitory factor (MIF) [45]. The high glucose levels that occur in insulin resistance states, oxidize the low-density lipoproteins which in turn have been shown to activate NF-Kβ and induce MIF expression, in vascular smooth muscle cells [46].

NFKβ is a drug target for drugs designed to reduce inflammatory processes associated with type 2 diabetes [47]. In a first step NFKβ is related to TRAF6 and KAT5. KAT5 is the catalytic subunit of NuA4 histone acetyltransferase complex that is involved in the acetylation of nucleosomal histone H4 and H2A producing an activation of transcription of selected genes. No previous relation with bodies or lipid metabolism has been identified for KAT5. On the other hand, a recent publication has shown that TRAF6, an adapter protein for the TNF receptor and interleukin-1R receptor, has a role in immunity by modulating fatty acid metabolism [48]. These set of relations offer another feasible way by which ketone bodies palliate the effects of elevated insulin resistance states.

CBL is an E3 ubiquitin-protein ligase involved on glucose uptake stimulated by insulin. Its inhibition is associated with insulin resistance [49]. We also found a direct interaction of CBL with TRAF6, which in turn links CBL with ACAT1 and ACAT2, enzymes of lipid metabolism involved on the fatty acids metabolism and the synthesis and degradation of ketone bodies.

Finally, it is worth stressing that neither of the hypotheses raised by our approach relates the beneficial effects described in type 2 diabetes with the significant weight loss achieved as a consequence of the diet itself, which correlates well with clinical observations that uncouple these two effects, since the improvements in the glycemic state are detected before a significant reduction of weight.

## Conclusions

The global molecular network analysis of conditions, diseases and treatments (either pharmacological or based in changes in life style) are bound to provide new insights and help in a better understanding of clinical, metabolic, and molecular relationships between them. The analysis of the ketogenic-diet map from the diabetes perspective offers interesting results and insights on the mechanism of action, but also opens new possibilities to study the applications of the ketogenic diet in other situations such as CNS or other metabolic dysfunctions. The strong relationship identified in our studies between the insulin resistance pathway and the ketogenic diet provides a plausible explanation for the improvement observed on clinical trials from a new and different perspective. Moreover, the map analysis highlights two complementary hypothesis of functional relationship between the molecules involved in both physiological processes: a) Elements of lipid metabolism may facilitate proper cellular localization of glucose transporter and recycling and b) Ketone bodies can alleviate certain inflammatory processes by blocking specific cytokines. Although our studies incorporate the most actual data on the field, this is a fast evolving field thus additional or complementary mechanisms can come into view as novel data on this physiological condition emerges.

The characterization and analyses of protein networks is indeed becoming a cornerstone in modern biology, with clear biomedical applications [4,5,50,51]. However, network and systems biology approaches are still in their infancy and will have to certainly overcome many caveats, mostly related to the quality of the data and its interpretation. For instance, we can assess the reliability of the relationship between molecules that have been described but, obviously, cannot say anything about the many thousands that are yet to be discovered. On the other hand, to be most valuable, the functional hints coming from global analyses, such as the potential relationships reported in this study, will need to be individually validated. We anticipate that large international efforts, such as the ongoing initiatives to chart disease-related interaction maps [52], will soon permit to generate the basic wiring inherent to most physio-pathological processes and refine systems biology models to the point where they can be effectively applied to biomedicine.

## Competing interests

JLR and PA have no financial relationships to disclose. JF, AP, MC, JN and JMM from Anaxomics have financial competing interests. The research conducted at Anaxomics has been prompted by PronoKal. AM is a consultant to PronoKal. IS is medical adviser of PronoKal and AF is the chairman of PronoKal.

## Authors' contributions

JF conceived of the study, and participated in its design and coordination and helped to draft the manuscript. JMM participated in the design of system biology approach. AP and JLR carried out the system biology studies. JN and MC participated in results interpretation and helped on draft the manuscript. AM and JF participated on the design of the study and on the physiological and medical interpretation of the results. PA designed the entire study and supervised all portions of the study. All authors read and approved the final manuscript.
